# Case Report: Fabry disease mimicking coronary artery disease and hypertrophic cardiomyopathy—a 15-year diagnostic delay

**DOI:** 10.3389/fcvm.2026.1846913

**Published:** 2026-06-18

**Authors:** Tielang Liang, Hongyuan Xu, Chuang Huang

**Affiliations:** Cardiology Department, The Eighth Affiliated Hospital of Guangxi Medical University, Guangxi, China

**Keywords:** enzyme replacement therapy, Fabry disease, gene, multi-system involvement, myocardial hypertrophy

## Abstract

**Background:**

Fabry disease is a rare X-linked hereditary lysosomal storage disorder. Its cardiac manifestations often overlap with those of hypertrophic cardiomyopathy or coronary artery disease, leading to significant delays in diagnosis.

**Case summary:**

A 60-year-old male patient presented with exertional angina pectoris, which had persisted for 15 years. Initially diagnosed with coronary artery disease, he underwent percutaneous coronary intervention. Despite successful revascularization, he subsequently developed progressive left ventricular hypertrophy, heart failure, bilateral hearing loss, dizziness, and white matter lesions in the brain. Cardiac magnetic resonance imaging revealed mid-myocardial striae-like late gadolinium enhancement and left ventricular high voltage on electrocardiography, raising a strong suspicion of Fabry disease. Plasma α-galactosidase A activity was significantly decreased (0.62 μmol/L), and genetic testing identified a hemizygous pathogenic variant in the *GLA* gene [c.718_719del (p.K240fs)], confirming the diagnosis of Fabry disease. Due to various reasons, enzyme replacement therapy was not initiated, and the patient is currently managed with a heart failure and secondary prevention regimen for coronary artery disease, with stable disease status.

**Discussion:**

This case highlights the challenges in identifying Fabry disease in patients with coexisting coronary artery disease and left ventricular hypertrophy. It emphasizes the diagnostic value of multi-system involvement and characteristic imaging findings. A diagnostic pathway incorporating clinical warning signs, cardiac magnetic resonance imaging, enzymatic assays, and genetic testing can help reduce diagnostic delays.

## Introduction

Fabry disease (FD) is a rare X-linked lysosomal storage disorder with an estimated prevalence of 1/100,000 in the general population (1). It results from mutations in the *GLA* gene located at Xq22.1, leading to partial or complete deficiency of α-galactosidase A. This enzymatic defect causes progressive accumulation of its substrates—globotriaosylceramide (Gb3) and its derivative lyso-Gb3—in multiple tissues, including the kidneys, heart, nerves, and skin (2), ultimately driving multi-system dysfunction (3). The clinical spectrum is broad and heterogeneous, with classic features including episodic acroparesthesia, hypohidrosis, angiokeratomas, corneal verticillata, and proteinuria.

A particular phenotype exists in which cardiac involvement is the dominant or sole manifestation. This cardiac variant typically presents as left ventricular hypertrophy (LVH), frequently accompanied by conduction abnormalities and myocardial fibrosis (4). Onset tends to be later, most commonly between 40 and 60 years old, and classic symptoms such as neuropathic pain or cutaneous lesions are often absent. According to the 2023 Chinese Guidelines for the Diagnosis and Management of Hypertrophic Cardiomyopathy in Adults, among patients diagnosed with hypertrophic cardiomyopathy (HCM) after age 35, 0.5%–1.0% harbor underlying Fabry disease (5).

The clinical and imaging features of Fabry disease overlap substantially with more common conditions such as HCM, hypertensive heart disease, and even cardiac amyloidosis, making clinical recognition difficult. As a result, the average diagnostic delay exceeds 10 years. This delay frequently causes patients to miss the critical window for early initiation of enzyme replacement therapy (ERT), allowing irreversible progression of cardiac, cerebral, and renal damage and increasing the risk of heart failure, malignant arrhythmias, stroke, and death (6).

We report a case of Fabry disease that was missed for over a decade, aiming to raise clinicians' awareness of this rare disorder in the differential diagnosis of adult-onset LVH and provide a practical early diagnostic pathway to reduce misdiagnosis and missed diagnosis.

## Case description

### Chief complaint and history of presenting illness

A 60-year-old male patient was admitted to the hospital in December 2015 with recurrent exertional chest tightness and pain that had been ongoing for more than 5 years and had worsened significantly over the preceding 10 days. His medical history included hypothyroidism, for which he had been taking regular levothyroxine. He had no history of smoking, hypertension, or diabetes. His elder brother had a known history of myocardial hypertrophy.

The patient's clinical course was prolonged and characterized by gradual progression across several distinct phases, during which his treatment plan was repeatedly adjusted in response to emerging symptoms.

#### Phase 1: diagnosis of coronary heart disease with unidentified etiology of myocardial hypertrophy (2010–2015)

Around 2010, the patient began experiencing substernal, squeezing chest pain during physical activities such as brisk walking or climbing stairs. These episodes typically lasted 5–10 min and resolved completely with rest, a pattern consistent with typical exertional angina. To investigate this, he underwent coronary angiography at an outside hospital. The results showed a normal left main coronary artery, a 30%–40% focal stenosis in the proximal left anterior descending artery (LAD), and a 40% stenosis in the proximal right coronary artery. Because the stenoses were not considered severe enough to warrant intervention, he was started on secondary prevention medications for coronary artery disease. Despite this, his angina symptoms persisted intermittently.

In late December 2015, his angina episodes increased in frequency and severity, leading to another hospitalization. A repeat coronary angiogram revealed significant progression of the LAD lesion to 85% stenosis. Thus, the patient was diagnosed with coronary atherosclerotic heart disease. A drug-eluting stent was successfully implanted in the proximal LAD. An echocardiogram performed during this hospitalization provided the first indication of a significant underlying structural issue, showing a thickening of the interventricular septum and left ventricular posterior wall, with preserved left ventricular systolic function. The report also noted mild mitral regurgitation and did not rule out HCM. The patient was discharged on a regimen including aspirin, simvastatin, clopidogrel, trimetazidine, and levothyroxine. The finding of myocardial hypertrophy was not investigated further at that time.

#### Phase 2: diagnosis revised to hypertrophic cardiomyopathy due to progressive worsening of hypertrophy (2016–early 2024)

**Figure 1:**
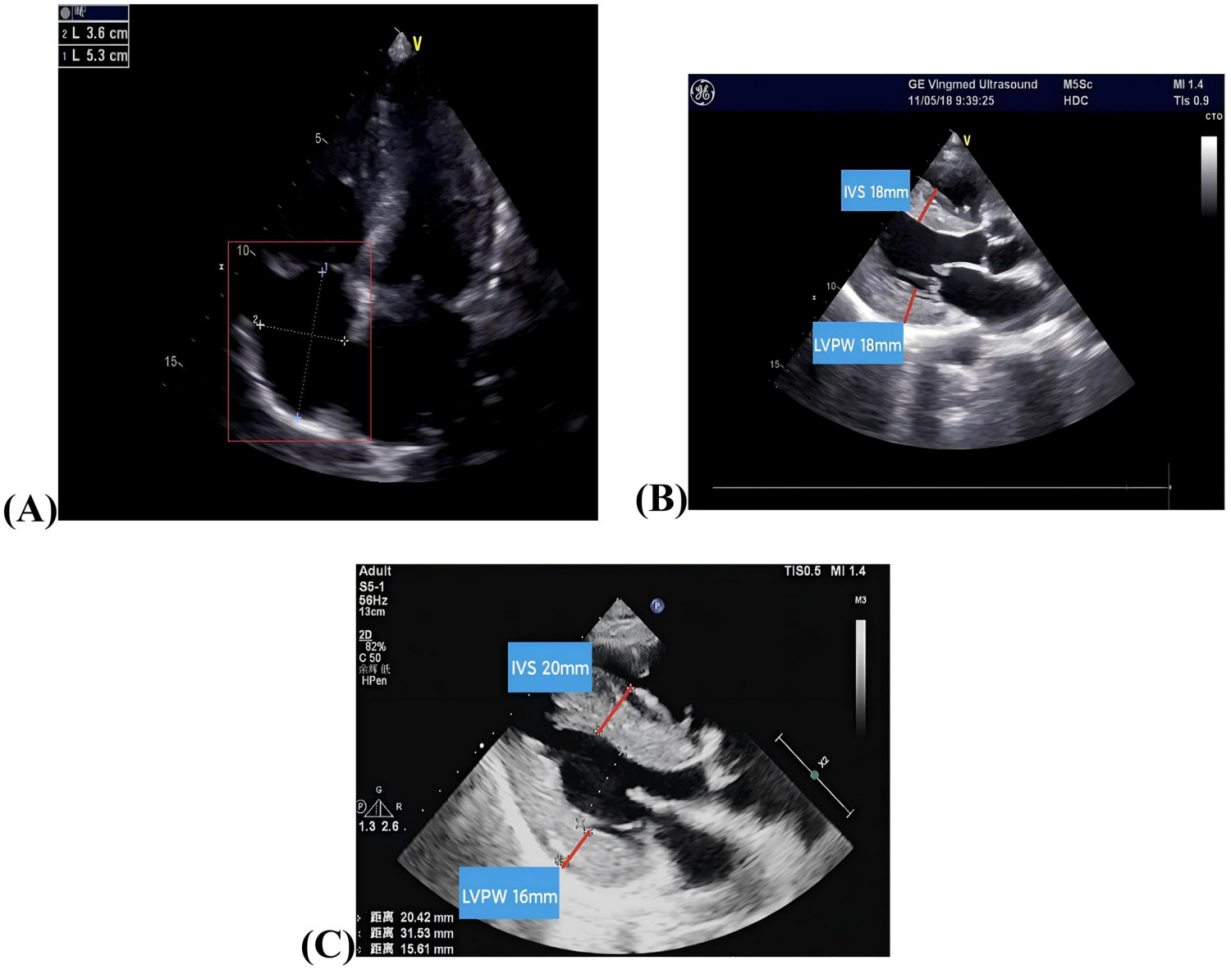
Dynamic echocardiographic changes in the patient with Fabry disease. **(A)** Echocardiography in 2018 shows symmetric left ventricular hypertrophy. **(B)** Echocardiography in 2018 shows interventricular septum and left ventricular posterior wall thicknesses, with both measuring 18 mm. **(C)** Echocardiography in 2025 shows septal thickness progression (basal septal thickness 20 mm).

In 2016, approximately 1 year after his stent placement, the patient developed exertional dyspnea and fatigue, which his physicians attributed to heart failure. Spironolactone (20 mg once daily) was added to his regimen, providing some symptomatic relief. A follow-up coronary angiogram in 2017 showed that the stent remained patent with no new significant stenoses. In 2018, the patient was re-evaluated for heart failure symptoms. An echocardiogram showed progression of his LVH (Figure 1A), with both the interventricular septum and posterior wall measuring 18 mm (Figure 1B). His left ventricular ejection fraction (LVEF) remained preserved at 67%. The pattern was moderate-to-severe symmetric hypertrophy, which could not be fully explained by ischemic heart disease or his underlying hypothyroidism. His diagnosis was revised to hypertrophic cardiomyopathy with chronic heart failure, and a loop diuretic, furosemide (20 mg once daily), was added to his treatment.

Beginning in 2022, the patient experienced intermittent vertigo and progressive bilateral hearing loss. A high-resolution computed tomography (CT) scan of his ears in 2023 suggested chronic suppurative otitis media in the right ear, but this finding did not adequately explain the bilateral nature of his hearing loss. Between 2023 and 2024, he was seen multiple times for dizziness, with outpatient evaluations treating it as posterior circulation ischemia with limited success.

In 2024, the patient was admitted to the neurology department due to frequent dizziness. An electrocardiogram (ECG) showed ST-segment depression, left ventricular high voltage, and signs of left atrial overload. Brain magnetic resonance imaging (MRI) revealed white matter hyperintensities in the bilateral frontal lobes and corona radiata. He was diagnosed with benign paroxysmal positional vertigo. Although the vertigo improved somewhat after canalith repositioning maneuvers and betahistine, the hearing loss and the white matter lesions continued to progress, and their underlying cause remained unclear.

#### Phase 3: imaging highly suggestive of infiltrative disease, suspected phenocopy cardiomyopathy (July—September 2024)

In July 2024, the patient's condition took an acute turn. Without any clear trigger, he developed bilateral, symmetrical, pitting edema in his lower extremities, accompanied by significant worsening of chest tightness and dyspnea even at rest. When sleeping, he needed to be propped up on multiple pillows. His N-terminal pro-B-type natriuretic peptide (NT-proBNP) level was 7,326 pg/mL.

The patient was referred to a tertiary care hospital for further evaluation. An echocardiogram revealed symmetric biventricular hypertrophy accompanied by characteristic increased myocardial echogenicity, significant biatrial enlargement, restrictive diastolic filling, left ventricular outflow tract obstruction, and a small pericardial effusion. The description of increased, dense myocardial echogenicity suggested a fundamental change in myocardial tissue texture, strongly indicating an infiltrative process such as cardiac amyloidosis. This was a pivotal clue.

To further characterize the myocardial pathology, cardiac magnetic resonance (CMR) imaging was performed. Morphological assessment revealed biventricular myocardial thickening, most prominent in the interventricular septum (10–19 mm), with mild free wall thickening (5–11 mm). The right atrium was dilated. Biventricular systolic function was mildly impaired (LVEF 51.37%, RVEF 53.07%), and no left ventricular outflow tract obstruction was detected. Myocardial perfusion imaging showed segmental linear perfusion defects in the basal posterior septum and inferior wall of the left ventricle. Late gadolinium enhancement (LGE) demonstrated arc-shaped subendocardial enhancement in the basal posterior septum and inferior wall, as well as focal mid-wall patchy and streak-like enhancement in the free walls of both ventricles. The CMR findings were initially interpreted as obstructive biventricular hypertrophic cardiomyopathy combined with ischemic heart disease. However, given the atypical imaging features and clues suggestive of a phenocopy, genetic testing was warranted to differentiate hypertrophic cardiomyopathy, cardiac amyloidosis, and dilated cardiomyopathy.

#### Phase 4: clear evidence of multi-system involvement, final diagnosis: Fabry disease (September 2024 onwards)

Given the combination of progressive cardiac disease with severe LVH and characteristic imaging findings, neurological involvement with white matter lesions, and auditory impairment with progressive hearing loss, a strong suspicion for FD arose.

**Figure 2:**
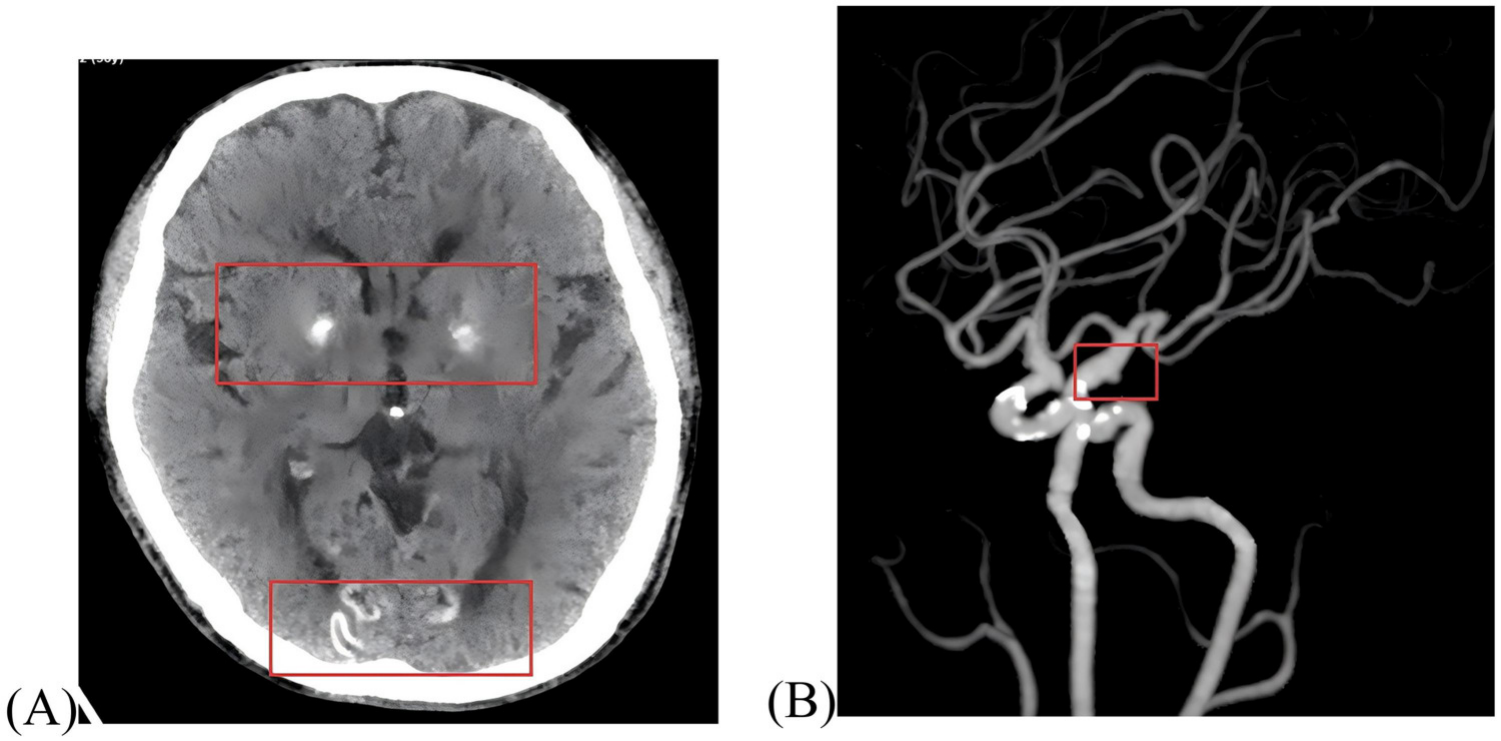
Head and neck computed tomography angiography (CTA) in the patient with Fabry disease. **(A)** Symmetrical intracranial calcifications involving the bilateral cerebellar hemispheres, basal ganglia, and occipital lobes. **(B)** A suspected intracranial aneurysm in the C7 segment of the right internal carotid artery.

In September 2024, enzyme activity testing was performed. The patient's plasma α-galactosidase A activity was markedly reduced at 0.62 μmol/L (Table 1A), compared with a normal reference range lower limit of 2.2 μmol/L. Subsequent *GLA* gene sequencing identified a hemizygous pathogenic variant, c.718_719del (p.K240fs) (Table 1B), confirming the diagnosis of FD. Regarding renal function assessment, the patient's serum creatinine was 95 μmol/L, with an estimated glomerular filtration rate of 79.5 mL/min/1.73 m^2^, indicating mild renal impairment (chronic kidney disease stage 2). In February 2025, a CT angiogram of the head and neck revealed bilateral, symmetrical calcifications in the basal ganglia and cerebellum (Figure 1A), as well as multiple lacunar infarcts and an internal carotid artery aneurysm (Figure 2B), further confirming cerebrovascular involvement. In Fabry disease, widespread sphingolipid deposition causes direct injury to cerebral microvascular endothelium, cranial nerves, and brain parenchyma, leading to persistent and progressive multi-site damage. Temporal bone pathology studies in patients with Fabry disease further confirm that glycosphingolipid accumulation within cochlear vascular endothelium results in chronic microvascular insufficiency and irreversible inner ear injury (7), which accounts for the patient's cerebral white matter lesions, lacunar infarcts, vertigo, and hearing loss. After excluding hypertensive small vessel disease, multiple sclerosis, central nervous system infections, and autoimmune encephalopathy, the neurological involvement further corroborates the diagnosis of Fabry disease.

**Table 1:** Laboratory confirmation of Fabry disease.

(A) Plasma α-galactosidase A activity			
Test item	Abbreviation	Result	Reference range
α-Gal A	CLA	0.62	2.20–17.65

**Table:** 

(B) *GLA* gene sequencing result							
Gene and transcript	Associated disease and inheritance pattern	Chromosomal location	Variant	Exon/intron	Zygosity	Variant classification	Variant origin
*GLA* NM 000169.3	Fabry disease (XL)	chrX：100653855-100653856	c.718_719del (p.K240fs)	Exon5	Hemizygous	Pathogenic	–

After integrating the patient's multi-systemic symptoms, characteristic cardiac imaging findings, positive family history, significantly reduced enzyme activity, and confirmed genetic mutation, he received a definitive diagnosis of FD, cardiac variant. ERT was strongly recommended. However, after being informed of the treatment details, the patient declined to initiate it at that time. After the patient declined ERT, the treatment team re-evaluated alternative targeted options. Pharmacological chaperone therapy was ruled out as it is only applicable in patients with Fabry disease who have certain missense mutations (8). Gene and substrate reduction therapies remain under clinical investigation and were not considered feasible at this time. The patient's current treatment regimen focuses on managing heart failure symptoms, cardiovascular risk, and complications related to coronary artery disease. This includes rosuvastatin (10 mg once nightly), clopidogrel (75 mg once daily), dapagliflozin (10 mg once daily), and mecobalamin (0.5 mg three times daily), along with sacubitril/valsartan, the dosage of which is adjusted between 25 mg and 50 mg once or twice daily based on his blood pressure.

### Follow-up

During long-term follow-up, the patient's blood lipids, glucose, and blood pressure remained generally well controlled, with no significant abnormalities. At the most recent follow-up, approximately 1 year after diagnosis, the patient had not experienced any further episodes of typical angina or acute decompensated heart failure. His New York Heart Association (NYHA) functional class improved to Class II (Table 2A), with mild limitation in physical activity. Moreover, his blood pressure was well-controlled at 105–110 over 70–80 mmHg, with a resting heart rate of 55–65 beats per minute, and his NT-proBNP level had significantly decreased to 1,292 pg/mL (Table 2B). A recent echocardiogram showed that the basal interventricular septum thickness remained at 20 mm (Figure 1C), his LVEF was stable at 68%, and the E/e' ratio was 12. His condition is currently stable, with the comprehensive heart failure management strategy successfully maintaining his cardiac function.

**Table 2:** Changes in key indicators at different time points in the patient with Fabry disease.

(A) NT-proBNP concentrations at different time points in the patient with Fabry disease			
July, 2024	December, 2024	February, 2025	December, 2025
7,326.00	10,335.00	6,843.00	1,292.6

Method: Chemiluminescence immunoassay; Unit: pg/mL.

**Table:** 

(B) NYHA functional class and symptom changes at different time points in the patient with Fabry disease		
Time point	NYHA functional class	Symptoms and degree of activity limitation
Early stage (before 2015)	Class I	No obvious discomfort during daily activities; no limitation in physical activity
Mid-course (2015–2023)	Classes II–III	Post-exertional chest tightness and dyspnea progressively worsened; daily physical activity was persistently limited
Acute exacerbation (July 2024)	Class IV	Dyspnea was present even at rest; unable to perform any physical activity
Recent follow-up	Class II	Symptoms significantly improved; mild limitation in physical activity

## Discussion

FD is classified into classic and late-onset phenotypes. Both phenotypes can occur in male and female patients. Male patients carry only one X chromosome, so the hemizygous state typically leaves them with near-total loss of enzyme activity—this is why they more often present with the classic phenotype, which is more severe and starts earlier (9). Female patients, by contrast, have a second X chromosome that can partially compensate, so their symptoms tend to be milder and show up later. It 's also worth noting that several factors can skew reported prevalence, including variable phenotypic penetrance, the presence of modifier genes, and the fact that female patients are underdiagnosed (10). The present case aligns with late-onset FD, presenting in middle age with angina, followed by progressive LVH, heart failure, and multi-systemic manifestations including vertigo, hearing loss, and white matter lesions.

The patient's diagnostic journey was lengthy, shifting from coronary artery disease to HCM and heart failure, then to suspicion of phenocopy cardiomyopathy, before finally reaching the correct diagnosis. The initial echocardiogram revealed symmetric LVH not explained by coronary disease alone—an early warning sign that was not pursued. The subsequent emergence of neurological and auditory symptoms provided crucial clues pointing to a systemic disorder, necessitating a multi-system rather than organ-specific perspective.

In the differential diagnosis of LVH, a systematic approach is essential. Post-load causes such as hypertension and aortic stenosis should first be excluded. The patient had no history of hypertension, and his aortic valve was structurally normal; these were ruled out. Next, primary HCM was considered. HCM typically presents with asymmetric hypertrophy, patchy LGE on CMR, and is confined to the heart. In contrast, the patient's CMR revealed a characteristic mid-wall linear LGE pattern accompanied by multi-system involvement, findings inconsistent with HCM. A key differential was cardiac amyloidosis, which on CMR often shows diffuse subendocardial LGE with significantly elevated T1 values and low voltage on electrocardiography. The findings in this patient, i.e., mid-wall LGE due to sphingolipid deposition and left ventricular high voltage on ECG, were not consistent with amyloidosis. When common etiologies are excluded and multi-system symptoms are present, inherited metabolic disorders such as Fabry disease should be prioritized in the differential diagnosis.

Minimally invasive interventional and non-invasive imaging findings served as crucial triggers for initiating specific enzymatic and genetic testing. Serial echocardiography in this case documented progressive LVH, along with the emergence of increased myocardial echogenicity, biatrial enlargement, diastolic dysfunction, and outflow tract obstruction. CMR provided critical diagnostic clues. Mid-wall linear LGE is a characteristic feature of cardiac involvement in Fabry disease, distinguishing it from HCM or cardiac amyloidosis. Notably, early coronary angiography revealed only mild-to-moderate stenosis, yet the patient already presented with typical exertional angina symptoms disproportionate to the degree of luminal narrowing. This discrepancy suggests Fabry disease-related coronary microvascular dysfunction or vasospasm as the likely primary mechanism (11). However, the subsequent progression to an 85% obstructive lesion necessitating stent implantation indicates that later-stage disease followed the natural history of atherosclerotic pathology. Accordingly, this case is best understood as two coexisting processes in a patient with Fabry disease, i.e., functionally driven angina early on, attributable to microvascular dysfunction or spasm, with superimposed atherosclerotic obstructive disease emerging later. This clinical trajectory also raises the following important question: Do the metabolic derangements inherent in Fabry disease accelerate atherogenesis? Further research is needed to address this.

For suspected cases, plasma α-galactosidase A activity is a sensitive screening test, with confirmation by *GLA* gene sequencing. In male patients, severely reduced activity is strongly indicative of FD (12); however, due to X-chromosome inactivation, over 60% of female patients have enzyme activity within the normal range (13). The variant carried by this patient, c.718_719del (p.K240fs), is a frameshift mutation located in exon 6 of the *GLA* gene. The deletion of two adenine bases shifts the reading frame, introducing a premature stop codon after lysine 240. The resulting truncated α-galactosidase A lacks the intact catalytic domain, with near-complete loss of enzymatic activity, leading to marked systemic accumulation of Gb3 and lyso-Gb3. This variant has been reported in the Brazilian population (14) and is typically linked to the classic Fabry disease phenotype; it presents as a progressive multi-system disorder, characterized by acroparesthesia, hypohidrosis, angiokeratomas, corneal verticillata, progressive renal insufficiency, cardiomyopathy, and cerebrovascular events. However, subsequent studies have confirmed that even patients carrying the same pathogenic variant can show marked phenotypic heterogeneity (15). That is exactly what we see here—an atypical, late-onset multi-system presentation dominated by cardiac involvement, with clear neurological and auditory system involvement as well.

As an X-linked disorder, pathogenic *GLA* mutations can be transmitted to offspring. Cascade family screening is essential once a proband is diagnosed, enabling early identification of affected individuals (16, 17). The patient's elder brother has a history of myocardial hypertrophy, suggesting disease clustering; first-degree relatives should undergo genetic testing and clinical evaluation. We did recommend screening to the patient and his elder brother; however, given the brother's lack of overt symptoms and financial constraints, he declined further clinical evaluation and genetic testing.

Management includes supportive care and disease-specific therapy, such as ERT, chaperone therapy, and gene therapy (18, 19). ERT, which provides exogenous recombinant α-galactosidase A, is the first-line disease-specific treatment (20) and can reduce left ventricular mass (21). In this case, the patient declined ERT due to financial constraints, insurance limitations, personal concerns, and access challenges—highlighting that treatment initiation is influenced not only by clinical factors but also by socioeconomic and healthcare system factors.

## Conclusion

It 's worth highlighting that the symptoms of Fabry disease overlap heavily with far more common conditions—hypertensive heart disease, hypertrophic cardiomyopathy, and the like—so missed diagnoses are a real problem in clinical practice. Therefore, proactive screening and long-term monitoring of high-risk populations must be strengthened, as they are the key to shortening diagnostic delay and ensuring early treatment. Thus, clinicians should consider Fabry disease as part of the routine workup whenever they encounter left ventricular hypertrophy. Based on this case experience, we propose the following “Screening–Diagnosis–Management” clinical pathway: (1) screen when red flags are present (unexplained LVH with characteristic CMR features, neurological manifestations, or family history); (2) confirm with enzyme and genetic testing, followed by comprehensive systemic evaluation; (3) manage with early ERT when feasible, supportive care, cascade family screening, and multidisciplinary follow-up. Early diagnosis through this structured approach can prevent irreversible organ damage and improve outcomes.

## Data Availability

The datasets presented in this article are not readily available because of patient privacy. Requests to access the datasets should be directed to Hong-Yuan Xu at xuhongyuan1239@163.com.
